# Target-Guided Isolation and Purification of Antioxidants from *Urtica laetevirens* Maxim. by HSCCC Combined with Online DPPH-HPLC Analysis

**DOI:** 10.3390/molecules28217332

**Published:** 2023-10-29

**Authors:** Aijing Li, Mencuo La, Huichun Wang, Jianzhong Zhao, Yao Wang, Ruisha Mian, Fangfang He, Yuhan Wang, Tingqin Yang, Denglang Zou

**Affiliations:** 1 School of Life Science, Qinghai Normal University, Xining 810008, China; liaijing21@mails.ucas.ac.cn (A.L.); m15349771021@163.com (M.L.); wwwyao9@163.com (Y.W.); 13369703315@163.com (R.M.); 15859557623@163.com (F.H.); wx28465@sina.com (Y.W.); ytq2022128@outlook.com (T.Y.); 2 Agricultural and Rural Science and Technology Guidance Development Service Center of Qinghai Province, Xining 810008, China; aylin1030@163.com

**Keywords:** *Urtica laetevirens* Maxim., online HPLC-DPPH, HSCCC, antioxidant active molecules, response surface methodology

## Abstract

*Urtica laetevirens* Maxim. is used extensively in traditional Chinese medicine (TCM) for its potent antioxidative properties. In this study, three antioxidants were purified from *U. laetevirens.* using HSCCC guided by online DPPH-HPLC analysis. Firstly, the online DPPH-HPLC analysis was performed to profile out the antioxidant active molecules in *U. laetevirens.* The ultrasonic-assisted extraction conditions were optimized by response surface methodology and the results showed the targeted antioxidant active molecules could be well enriched under the optimized extraction conditions. Then, the antioxidant active molecules were separated by high-speed countercurrent chromatography ethyl acetate/*n*-butanol/water (2:3:5, *v*/*v*/*v*) as the solvent system. Finally, the three targets including 16.8 mg of Isovitexin, 9.8 mg of Isoorientin, and 26.7 mg of Apigenin-6,8-di-C-β-d-glucopyranoside were obtained from 100 mg of sample. Their structures were identified by ^1^H NMR spectroscopy.

## 1. Introduction

*Urtica laetevirens* Maxim. (*U. laetevirens*) holds a distinguished place both in culinary traditions and in the field of natural medicinal plants, attributed to its potent antioxidative attributes [1]. Recognized as a rich repository of diverse nutritional and bioactive compounds, *U. laetevirens* aligns well with the burgeoning demand for antioxidant-rich foods, a demand propelled by a health-centric approach prevalent in contemporary dietary practices [2]. In this milieu, it is observed that the chemical constituents in *U. laetevirens* function effectively as free radical inhibitors, presenting themselves as primary antioxidants capable of reacting with free radicals [3]. This interactivity not only establishes a protective barrier against free-radical-induced damage within the human body but also augments the health benefits attributable to the consumption of *U. laetevirens* [4]. Consequently, there is an emergent imperative for the free radical inhibitors within *U. laetevirens* to be identified and segregated. In this study, an effort is made to locate and isolate the active constituents that exhibit significant antioxidative properties in *U. laetevirens*. Through such a systematic approach, the comprehensive antioxidant potential of *U. laetevirens* is envisioned to be revealed, paving the way for a wider and better *U. laetevirens* application in traditional herbs and dietary paradigms.

Diseases triggered by oxidative stress are notably linked with an extensive variety of severe health issues, encompassing cardiovascular ailments and various forms of cancer, consequently presenting a considerable menace to human health [5]. Within the rich molecules of *U. laetevirens*, bioactive constituents have been discovered to act as free radical inhibitors or scavengers, engaging actively with free radicals [6]. This interaction is critical in reducing the detrimental effects induced by oxidative stress in the human body. Leveraged for its reliability and effectiveness, the DPPH (1,1-diphenyl-2-trinitrophenylhydrazine radical) neutralization test facilitates the determination of the antioxidative potency of various agents, providing a further understanding of the role *U. laetevirens* plays in mitigating the risks associated with oxidative stress diseases [7]. The online HPLC (high-performance liquid chromatography)–DPPH screening system has emerged as a pivotal tool in the rapid identification of compounds with DPPH radical elimination potential in extracts [8]. The introduction of this online HPLC-DPPH technique has greatly streamlined the identification of active antioxidant constituents derived from natural sources, marking a substantial stride in the ongoing efforts to harness the potential of natural derivatives for antioxidative applications [9]. Furthermore, this technology has been demonstrated to be effective in the precise identification of antioxidants, asserting its utility and efficiency in the field of antioxidant research [10].

Response surface methodology (RSM) is acknowledged as a powerful instrument in fine-tuning the extraction procedures for active substances [11]. Leveraging precise mathematical and statistical techniques, it allows for the streamlined delineation of optimal conditions for extraction [12]. Moreover, it is extensively utilized to pinpoint the ideal conditions for operation [13,14,15,16]. Consequently, we utilized RSM in this study to refine the conditions for ultrasonic-assisted extraction.

Traditional methods of separation are often beset with constraints pertaining to yield purity and efficiency [17]. In comparison, high-speed countercurrent chromatography (HSCCC) stands out as a sophisticated alternative to traditional techniques, unencumbered by similar constraints, thereby offering enhanced accuracy and efficacy in segregating desired molecules [18]. Characterized by the employment of a liquid stationary phase rather than a solid one, it enables superior recovery rates and purity, representing a significant advancement in the realm of separation science [19].

In the present work, a framework has been meticulously devised for the swift and proficient delineation, isolation, and refinement of DPPH inhibitors in *U. laetevirens* using an integrated approach of online HPLC-DPPH analysis combined with HSCCC separation. This method not only showcases a strategic advancement in the rapid identification and isolation of antioxidative constituents but also fosters the discovery and application of antioxidants present in *U. laetevirens*, marking a pivotal step forward in the field of *U. laetevirens* chemical diversity and its health promotion effect.

## 2. Results and Discussion

### 2.1. Screening Target Antioxidants via the Online DPPH-HPLC System

We employed an online HPLC-DPPH approach to screen the antioxidant active molecules of *U. laetevirens*. This screening approach is highly regarded for its sensitivity and user-friendly operational characteristics, particularly well-suited for detecting antioxidant active molecules within a complex mixture [20]. The most salient advantage of the online post-column HPLC-DPPH system is its dual capacity to not only screen active molecules via UV detection but also simultaneously gauge the free radical scavenging activity [21]. Compounds exhibiting potential antioxidant activity manifest as corresponding negative peaks in the HPLC chromatogram at 517 nm after undergoing a DPPH reaction, while compounds devoid of antioxidant activity display minimal variations in peak areas [22].

**Figure 1:**
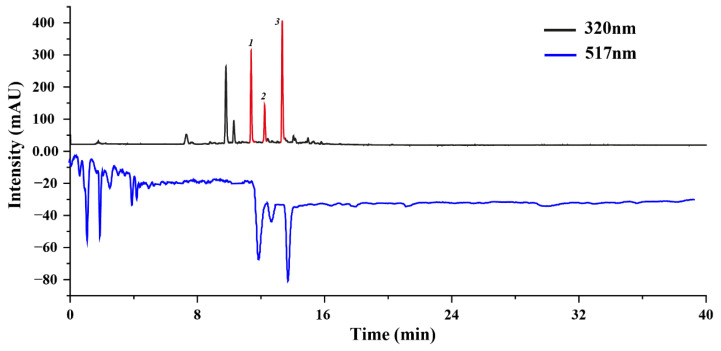
DPPH-HPLC analysis for antioxidant active molecule screen. Peak 1, 2, 3 were identified as target antioxidation active molecules. The HPLC settings were as follows: the analyses utilized a Phenomenex Luna C18 chromatographic column with dimensions of 250 mm × 4.6 mm and a particle size of 5 μm. The mobile phase was structured with component A (0.4% acetic acid dispersed in water) and component B (acetonitrile), following a pre-defined schedule: a time frame from 0 to 40 min was designated to facilitate a gradient increase from 5% to 95% of component B. The system operated at a steady flow rate of 1 mL/min. Detection was dual-wavelength, set at 320 nm and 517 nm, under a column thermostat maintained at 35 °C.

To initiate the analytical process, we utilized a Phenomenex Luna C18 chromatographic column within the online HPLC-DPPH system to profile DPPH inhibitors within the sample. Significantly, three well-resolved active peaks were discerned at 517 nm, precisely corresponding to the three chromatographic peaks detected at 320 nm in the HPLC chromatogram (Figure 1). Conversely, no other peaks exhibited commensurate active features. This observation substantiates the premise that these three peaks (Peak 1, 2, 3) are indicative of the target compounds, exhibiting potent antioxidant activity. Further experiments were performed to optimize the extraction efficiency of these target antioxidants through ultrasound-assisted extraction techniques, followed by the separation of these target antioxidants via HSCCC.

### 2.2. Refinement of Ultrasonic-Assisted Extraction Parameters

In adherence to the devised blueprint, a series of 17 experiments were executed twice to ensure reliability, with the garnered data illustrated in Table 1. The calculated values of R^2^, R^2^-adjusted, and R^2^-predicted were determined to be 95.64, 90.03, and 68.98, respectively, demonstrating that the comprehensive quadratic models offered superior efficacy in delineating the response variable Y compared to alternative models. The representative mathematical formulation is presented as:

(1) $$Y=60.79+3.07X_{1}+4.01X_{2}+0.6550X_{3}-5.206X_{1}X_{1}\;-5.02X_{2}X_{2}-1.95X_{3}X_{3}+2.36X_{1}X_{2}+1.41X_{1}X_{3}-0.51X_{3}\;\;\;\;\;$$

To scrutinize the substantiality of the model, an analysis of variance (ANOVA) was undertaken, the details of which are delineated in Table 2. In evaluating individual terms within the model, a heightened F-value paired with a diminished *p*-value signified a marked influence on the corresponding response element. Consequently, the linear constituents *X*_1_ and *X*_2_, the interactive term *X*_1×2_, along with the quadratic components *X*_1_^2^ and *X*_2_^2^, exhibited a considerable impact (*p* < 0.05) on the cumulative concentration of the three targets [23].

Contrastingly, the linear component X_3_, coupled with the interaction terms X_1×3_, and X_2×3_, and the quadratic element X_3_^2^, remained inconsequential in affecting the response, evincing a *p*-value greater than 0.05. The model’s predictive accuracy was confirmed by conducting a lack-of-fit assessment (*p* > 0.05), thus validating its capability to precisely predict encountered variances.

**Figure 2:**
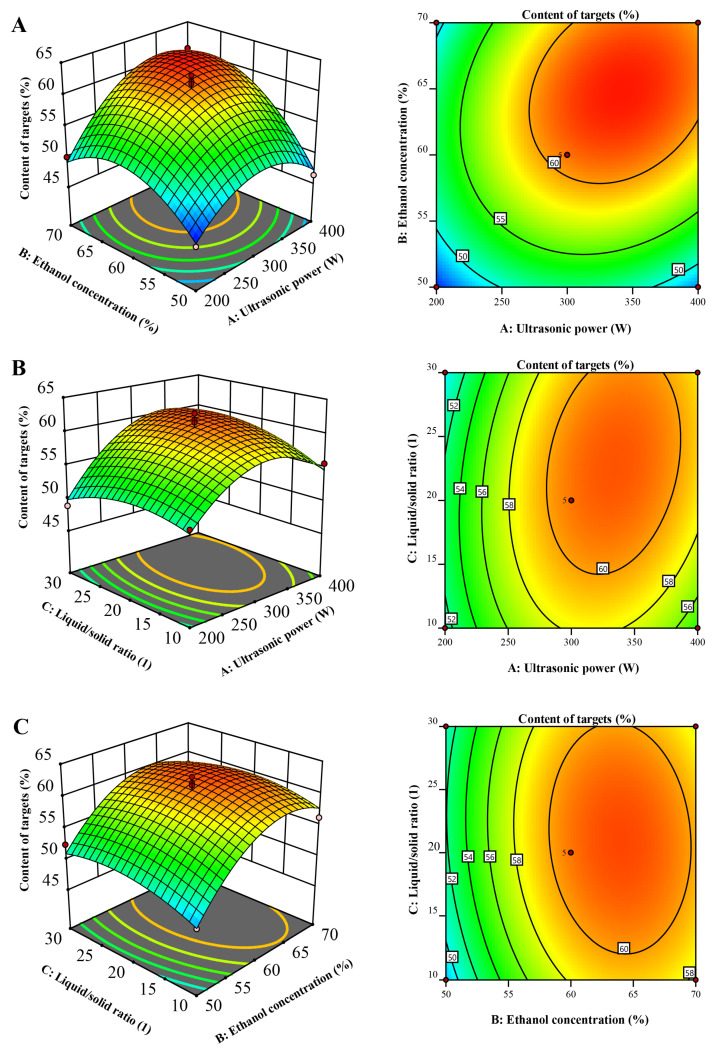
Response surface showing the interaction of variable factors on Y: (**A**) interaction between ultrasonic power and ethanol concentration; (**B**) ultrasonic potency in correlation with the liquid-to-solid ratio; (**C**) between ethanol concentration and the liquid-to-solid ratio.

The 3D diagrams serve as visual embodiments of the regression equations, facilitating the discernment of the associations between the outcomes and the experimental tiers of every variable, as well as delineating the interplays between a pair of testing parameters. These connections between independent and reliant variables have been graphically portrayed through three-dimensional renderings of the response surfaces designated for Y (illustrated in Figure 2). Each 3D surface plot illustrates two variables, with the remaining parameter maintained at a neutral level.

Figure 2A illustrates the correlation between ethanol concentration and ultrasonic power. Initially, there was an increase in Y with rising ethanol concentration, followed by a subsequent decrease. This phenomenon can be attributed to the disparity in polarities between the target compounds and other constituents. Within the optimal ethanol concentration range, the target compounds exhibited effective enrichment, similar to the performance of liquid–liquid extraction or macroporous resin chromatography. Consequently, Y exhibited an initial increase with a higher ethanol concentration. However, as the ethanol concentration continued to rise beyond the suitable range, the content of target compounds declined, leading to a subsequent decrease in W.

Figure 2B portrays the interaction between the liquid-to-solid ratio and ultrasonic power. Initially, there was an uptick in Y with the rising liquid-to-solid ratio, but subsequently, it commenced a descent. This phenomenon can be elucidated by considering the wide range of polarities within the sample. With an increase in the liquid-to-solid ratio, both the content of other compounds and target compounds simultaneously escalated. However, once the liquid-to-solid ratio exceeded 23, the relative growth rate of ΣCj (where j denotes the other compounds) outpaced that of ΣCi (where i represents the target compounds). Consequently, Y witnessed a decline.

The optimal conditions for the UAE process were determined to be an ultrasonic power of 344.16 W, an ethanol concentration of 64.89%, and a liquid/solid ratio of 23.64. The predicted Y under these conditions was 62.53%. Subsequently, the actual experimental parameters were set to a power of 345 W, an ethanol concentration of 65%, and a liquid/solid ratio of 23. These conditions were tested in triplicate, resulting in an achieved Y value of 61.63%. This outcome underscores the effectiveness of response surface methodology (RSM) with a well-designed experimental setup for optimizing the UAE process. These findings affirm that UAE is a viable choice for the extraction and concentration of the target compounds.

### 2.3. Determining the Appropriate Conditions for High-Speed Counter-Current Chromatography (HSCCC) Experiments

The initial stage of an HSCCC experiment involves carefully choosing a suitable solvent system that can provide the desired partition coefficient (K) for the target compounds, guided by their chemical properties [24]. Several crucial factors require examination, including the polarity of the sample (determined by K values), its solubility, ionic state, and potential for forming complexes [24]. Typically, an optimal K value falls within the range of 0.2 to 5, while the separation factor between two compounds (α = K2/K1, where K2 > K1) should exceed 1.5 [25]. Smaller K values orchestrate solutes to approach the solvent frontier, resulting in a lower resolution, whereas enlarged K values tend to offer superior resolution albeit with dilated and attenuated peaks due to protracted elution periods [26]. The prerequisite is the stability and solubility of target compounds, with a solvent system delineating briskly and distinctly into biphasic portions.

Heeding the stipulated principles, we streamlined the solvent scheme for HSCCC separation. We evaluated a range of two-phase solvent systems consisting of ethyl acetate/*n*-butanol/water (4:1:5, 3:1:5, 2:1:5, 3:2:5, 2:3:5, 1:4:5, *v*/*v*/*v*). We measured the K-values of the target compounds and have summarized the results in Table 3. Initially, a particular ratio of ethyl acetate/butanol/water (4:1:5, *v*/*v*/*v*) was leveraged to gauge the distribution of compounds. This revealed a predominant distribution in the lower phase, indicating a polarity less than what was necessitated by the sample. Consequently, adjustments were implemented on the ethyl acetate/butanol ratio to heighten the system’s polarity. The K values of the three targets exhibited a decline as the ratio of ethyl acetate to butanol decreased. Ultimately, we opted for the ethyl acetate/*n*-butanol/water system (2:3:5, *v*/*v*/*v*) due to its favorable K values and α-values.

Additionally, an array of flow rates was experimented with to unveil their repercussions on separation duration and peak discernment attributes. This revealed that diminished flow rates entailed extended separation times albeit with a commendable peak resolutions, the reverse being true for escalated flow rates. The ensuing choice was a 1.5 mL/min flow rate for ensuing HSCCC undertakings. Moreover, the rotational speed exhibited a conspicuous influence over stationary phase retention, with heightened speeds inciting emulsification, and elevated temperatures facilitating a higher retention in butanol inclusive solvent systems. A 900 rpm speed was hence adopted, attaining a 48% retention rate in the stationary phase.

**Figure 3:**
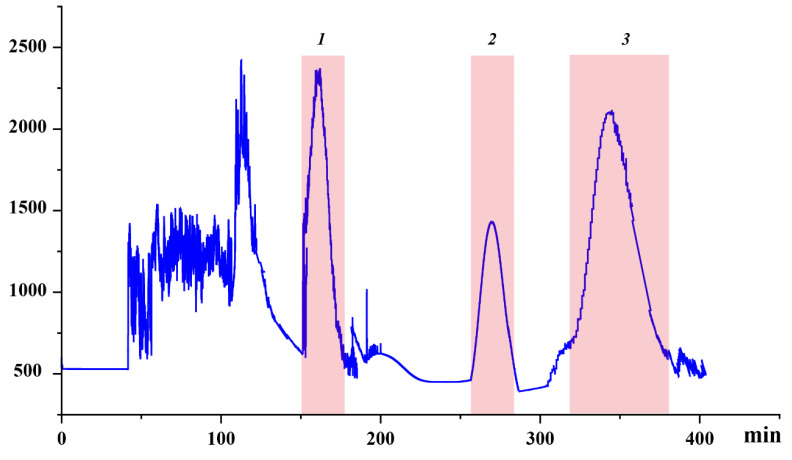
High-speed countercurrent chromatography (HSCCC) chromatogram of the sample using the ethyl acetate/*n*-butanol/water (2:3:5, *v*/*v*/*v*). Conditions: stationary phase, upper phase; flow rate, 1.5 mL/min; revolution speed, 900 rpm; sample amount, 100 mg; separation temperature, 40 °C; detection wavelength, 320 nm; retention of the stationary phase: 48%.

**Figure 4:**
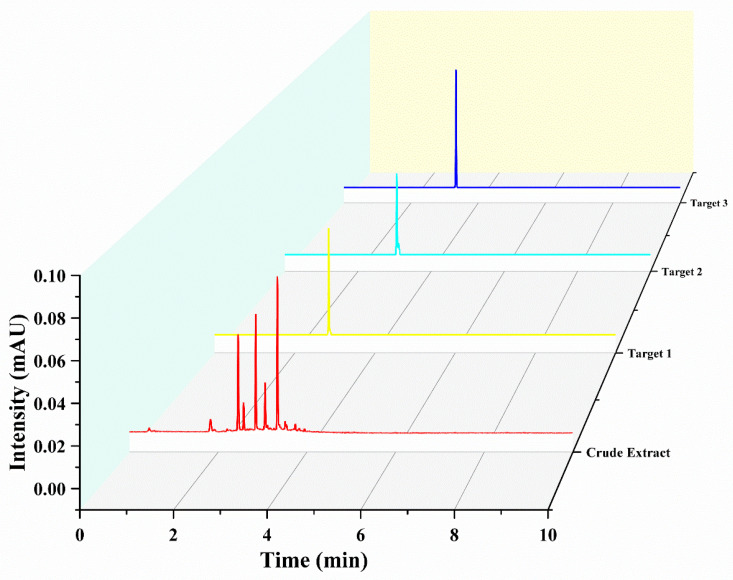
Ultra-high-performance liquid chromatography (UHPLC) chromatograms of both the raw extract and the fractions obtained through high-speed counter-current chromatography (HSCCC). Conditions: Separation was carried out using an Acquity UHPLCHSS T3 column (2.1 × 100 mm, 1.8 μm; Waters) at a maintained temperature of 35 °C. The delineated mobile phases included water supplemented with 0.1% formic acid (A) and acetonitrile (B). A gradient elution strategy was adopted, implementing a 5% to 95% B range over a span of 0–10 min, operating at a flow rate of 0.3 mL/min. The procedure involved an injection volume set at 1.0 μL. Throughout the process, a consistent column temperature of 35 °C was upheld, coupled with a detection wavelength pinpointed at 320 nm.

Under delineated parameters, a single run extracting from a defined quantity of rough extract in approximately 400 min yielded three principal antioxidants, as exhibited in Figure 3. Their quantities were detailed as 16.8 mg of Target 1, 9.8 mg of Target 2, and 26.7 mg of Target 3. UPLC analysis corroborated the purity of these entities to exceed 93%, as demonstrated in Figure 4.

### 2.4. Structural Identification

**Figure 5:**
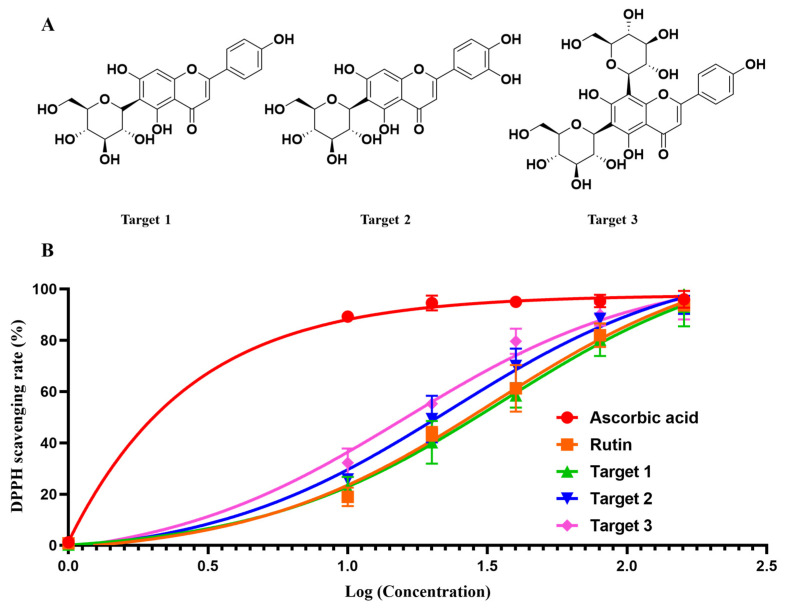
The chemical structures (**A**) and DPPH scavenging activity of the three targets (**B**): Isovitexin (Target 1), Isoorientin (Target 2), and apigenin-6,8-di-C-β-d-glucopyranoside (Target 3).

The chemical structures (Figure 5) of the target compounds were determined through ^1^H NMR analysis, and the findings are outlined below (Figures S1–S6, Supplementary Materials):

Target I: The compound was isolated as a pale yellow solid powder. Through meticulous nuclear magnetic resonance (NMR) spectroscopic analyses, its structure was elucidated. The ^1^H NMR (500 MHz, CD_3_OD) presented characteristic signals at δH: 4.59 (1H, t, *J* = 5.4 Hz, H-glc-1″), 6.51 (1H, s, H-3), 6.60 (1H, s, H-8), 6.93 (2H, d, *J* = 8.4 Hz, H-3′, 5′), and 7.83 (2H, d, *J* = 8.4 Hz, H-2′, 6′). The ^13^C NMR (500 MHz, CD_3_OD) provided additional data with δC values at: 62.84 (Glc-6″), 71.77 (Glc-4″), 72.56 (Glc-1″), 75.28 (Glc-3″), 80.12 (Glc-2″), 82.61 (Glc-5″), 95.25 (C-8), 103.87 (C-3), 105.19 (C-10), 109.20 (C-6), 117.06 (C-5′), 123.11 (C-1′), 129.46 (C-2′, 6′), 158.71 (C-9, C-3′), 162.04 (C-5), 162.79 (C-4′), 164.95 (C-2), 166.22 (C-7), and 184.06 (C-4). Comparisons of these spectroscopic data with literature values confirmed the identification of the compound as Isovitexin [27,28].

Target II: The compound, presenting as a pale yellow solid powder, was delineated through meticulous spectroscopic analyses. ^1^H NMR spectroscopy (500 MHz, CD_3_OD) divulged distinct resonances: δH 4.19 (d, *J* = 10.0 Hz, Glc-H-1″), 6.42 (s, H-3), 6.53 (s, H-8), 6.89 (d, *J* = 8.2 Hz, H-5′), 7.40 (dd, *J* = 8.2, 2.0 Hz, H-6′), and 7.44 (d, *J* = 2.0 Hz, H-2′). Concurrently, ^13^C NMR (500 MHz, CD_3_OD) featured δC values revealing the structural intricacies; notable peaks included: 62.83 (C-6″), 71.75 (C-4″), 72.48 (C-2″), 75.24 (C-1″), 80.01 (C-3″), 82.63 (C-5″), alongside others delineating the aromatic region and distinguishing the core structure. When juxtaposed with established literature, a notable consistency in the spectral data affirmed the compound’s identity as Isoorientin [20,29].

Target III: The compound manifested as a yellow powder was analyzed using high-resolution NMR spectroscopy. ^1^H NMR (500 MHz, DMSO-*d_6_*) revealed signals at δ: 8.01 (2H, d, *J* = 8.1 Hz, H-2′, 6′), 6.90 (2H, d, *J* = 8.3 Hz, H-3′, 5′), 6.80 (1H, s, H-3), in addition to resonances corresponding to the protons of 6-C-β-Glu and 8-C-β-Glu moieties at δ 4.78 (1H, d, *J* = 10.2 Hz, H-1′) and δ 4.81 (1H, d, *J* = 9.2 Hz, H-1′), respectively, and a multiplet spanning δ 3.22 to 3.87 associated with sugar protons. Further, ^13^C NMR (125 MHz, DMSO-*d_6_*) furnished characteristic carbon resonances for the aromatic ring at δ 164.7 (C-2), 104.2 (C-3), 183.5 (C-4), 158.9 (C-5), 107.8 (C-6), 161.1 (C-7), 106.1 (C-8), 156.5 (C-9), and 104.0 (C-10), coupled with the signals for the substituent groups which are illustrated in the ^1^H NMR data. Upon rigorous comparison with the existing literature data, the spectral details exhibited a congruence, thereby substantiating the identification of the compound as Apigenin-6,8-di-C-β-d-glucopyranoside [30,31].

### 2.5. DPPH Scavenging Activity Confimation

As shown in Figure 5B, the three targets exhibited satisfied DPPH scavenging activity, with IC_50_ values of 35.49 μg/mL, 23.36 μg/mL and 16.02 μg/mL, respectively. For the control, ascorbic acid gave an IC_50_ of 3.02 μg/mL and rutin afforded an IC_50_ of 33.03 μg/mL. Although the three targets gave a relatively higher IC_50_ than ascorbic acid, it was comparable to the known antioxidant rutin. The confirmation of the DPPH scavenging activity of the three targets revealed the effectiveness and applicability of our strategy for antioxidants’ identification and purification.

## 3. Materials and Methods

### 3.1. Equipment

The HPLC assessment was carried out utilizing the Agilent 1200 apparatus (Agilent Technologies Co., Ltd., Santa Clara, CA, USA), outfitted with components including a solvent distribution mechanism, a UV-VIS DAD sensor, column temperature controller, an automatic sample loader, and a chemstation. For the HSCCC experiments, we utilized the TBE-300B high-speed counter-current chromatography instrument (Shanghai Tauto Biotech, Shanghai, China). The apparatus featured a trio of preparative coils constructed from polytetrafluoroethylene, each boasting a capacity of 280 mL and an inner diameter measuring 1.6 mm. The setup also incorporated a 20 mL sampling loop for injecting samples. Positioned at various β values from 0.5 to 0.8, from the inner to the outer terminals respectively, the multi-tiered coil had a revolution radius of 5 cm. To manage the revolution speed ranging between 0 and 1000 rpm, a speed modulation device was integrated into the system. Further augmenting the setup were a steady-flow pump and a detection unit functioning at 320 nm, coupled with a N2000 workstation. The experiment utilized a thermal circulation system to maintain a consistent temperature throughout the process. Ultrasonic-assisted extraction research was conducted using a KQ-500E ultrasonic bath, which was procured from Kun Shan Ultrasonic Instruments Co., Ltd. (Jiangsu, China). The HPLC-DPPH analyses were effectuated through an Agilent 1200 mechanism provided by Agilent Technologies, outfitted with a collection of elements including a DAD detector, an automatic sampler, and a solvent distribution unit. The system operates under the guidance of ChemStation software (Version No. Rev. B. 04. 02 SP1) and utilizes a Phenomenex Luna C18 chromatography column (250 mm × 4.6 mm, 5 μm) to accomplish separation procedures. In regard to spectral analyses, a Bruker 400 MHz NMR spectrometer was engaged to facilitate the study.

### 3.2. Reagents and Plant Material

Every chemical agent used during the extraction and purification processes was of analytical quality, sourced from Jinan Reagent Factory. For the HPLC assessments, we employed chromatographically pure acetonitrile and formic acid, both obtained from CNW (Anpel Laboratory Technologies Inc., Shanghai, China). The investigations consistently employed deionized water at every stage. The DPPH was sourced from Sigma-Aldrich, a subsidiary of the Merck Group based in Germany. *U. laetevirens* incorporated in the analysis were acquired from a reliable supplier, following which they were subjected to drying and pulverization to a fine powder to streamline subsequent analytical processes.

### 3.3. HPLC and On-Line HPLC-DPPH Analysis

High-performance liquid chromatography (HPLC), alongside online HPLC-DPPH evaluations, were carried out utilizing a Phenomenex Luna C18 analytical column (250 mm × 4.6 mm, 5 μm). The flowing phase involved a mixture of water infused with 0.1% formic acid (component A) and acetonitrile (component B), following a specific gradient elution schedule detailed below: a time span from 0 to 40 min guided by a 5–95% B gradient, all while sustaining a 1 mL/min flow velocity. We designated a 10 μL quantity for injection, preserving a steady column warmth of 35 °C and establishing the detection wavelength at 320 nm.

For the online HPLC-DPPH analysis, the conditions remained largely consistent with the HPLC parameters described. For the subsequent column alteration process, a methanol-dissolved DPPH solution with a concentration of 50 μg/mL was utilized, maintaining a flow velocity of 0.5 mL/min, while tracking the absorbance at a wavelength of 517 nm.

### 3.4. Optimization of Ultrasound-Assisted Extraction Conditions

The optimization of ultrasonic-assisted extraction (UAE) parameters for the retrieval of desired compounds was conducted using response surface methodology (RSM). This approach leveraged a Box–Behnken experimental layout, considering three autonomous variables.

In common practice for UAE extraction optimization through RSM, several factors including ultrasonic power, solvent concentration, the liquid-to-solid ratio, and extraction time are taken into consideration. The extraction time poses a particular challenge; although longer durations potentially yield higher extraction rates, identifying the optimal time point—where the majority of targets are extracted and subsequent extraction offers diminishing returns—is non-trivial. This led to the choice of three critical variables for this study: ultrasonic power (*X*_1_), ethanol concentration (*X*_2_), and liquid-to-solid ratio (*X*_3_).

The aggregated concentration of the target elements, denoted by Y (where Y equals the total of Ci, with Ci signifying the percentage of the peak area corresponding to each targeted component), was selected as the response metric. The scheme of experimentation comprised 17 iterations, incorporating 12 factorial coordinates along with 5 core coordinates to enable experiment replication, elucidated in Table 1. In order to mitigate the influences of unpredicted fluctuations stemming from systematic inaccuracies, a randomized order was employed for conducting the experiments.

To analyze the relationships between the independent and dependent variables, a multiple regression analysis employing the least squares method was conducted. This facilitated the fitting of the experimental data to a second-order polynomial equation, the form of which is presented below:

(2) $$Y=\beta_{0}+\beta_{1}X_{1}+\beta_{2}X_{2}+\beta_{3}X_{3}+\beta_{11}X_{1}X_{1}\;+\beta_{22}X_{2}X_{2}+\beta_{33}X_{3}X_{3}+\beta_{12}X_{1}X_{2}+\beta_{13}X_{1}X_{3}+\beta_{23}X_{2}X_{3}$$

Within this framework, “*Y*” signifies the projected outcome, while *β*_0_ is the model’s intercept. The coefficients *β*_1_, *β_2_*, *β*_3_ pertain to the linear elements, *β*_11_, *β*_22_, *β*_33_ are linked with quadratic elements, and *β*_12_, *β*_13_, *β*_23_ are indicative of the interactive elements concerning the independent variables denoted as *X*_1_, *X*_2_, and *X*_3_ in the evaluation [12].

The effectiveness and suitability of the formulated models were evaluated through comprehensive analysis employing various metrics: the determination coefficient (R^2^), along with its modified (R^2^-adj) and forecasted (R^2^-pred) variants.

The R^2^ metric, fluctuating in a range from 0 to 1, functions as a tool to measure the precision of the model, demonstrating greater reliability as it nears 1. This figure is derived from the ratio of the regression sum of squares to the complete sum of squares, illustrating the percentage of data fluctuation represented by the model.

Moreover, the elevated figures of both the adjusted and forecasted determinants of coefficient (R^2^-adj and R^2^-pred) indicate a commendable alignment of the model with the acquired data set.

To guarantee the selection of the most precise model, a careful choice was pursued, succeeded by an examination employing the ANOVA technique, which evaluated the regression coefficients for statistical relevance using Fisher’s F-test with a 95% confidence interval.

Finally, to visualize the interactive implications of the variables, surface plots were generated based on the selected model, serving to delineate the intricate relationships among the influential factors.

### 3.5. Ultrasonic-Assisted Extraction

Dried *U. laetevirens* weighing 1 kg was pulverized into a fine powder and subjected to three UAE extraction processes utilizing 65% ethanol at a power setting of 345 W and a liquid-to-material ratio of 23. Following the extraction, all the filtrates were amalgamated and desiccated under vacuum conditions, yielding 112 g of crude extract, and the final target yield was 61.63%.

### 3.6. Determining the Two-Phase Solvent System

The suitable biphasic solvent framework was determined in consideration of the partition coefficient (K) pertaining to the distinct components targeted in the specimen. To ascertain the K metric, a standardized HPLC technique was utilized: a sufficient amount of raw sample dust was diluted in a beforehand balanced biphasic solvent environment, followed by its transfer to a segregation flask. Following a robust agitation ensuring a full balance of the biphasic specimen, a 2 mL portion from every phase was dried to remove all moisture. Afterwards, the remnants were reconstituted in 1 mL of methanol for subsequent HPLC scrutiny [32]. The K metric was defined as the proportion of the peak area associated with the target compound in stationary phase to that in mobile phase [22].

### 3.7. Preparation of Biphasic Solvent System and Sample Solution

The biphasic solvent system utilized in this experiment was formulated using a mixture of ethyl acetate/*n*-butanol/water, following a volumetric ratio of 2:3:5, respectively. This mixture was transferred into a separating funnel where it was allowed to reach a state of equilibrium at ambient temperature. Subsequent to this, the upper and lower phases were distinctly isolated. To remove any dissolved gases, both phases underwent a degassing process in an ultrasonic bath for a duration of 30 min, executed shortly before initiating the experiment.

To ready the test liquid for HSCCC separation, 100 mg of the crude extract in its desiccated powdered state was utilized. It was then evenly dispersed in 10 mL of a solvent blend constituted of equal portions (5 mL each) of the upper and lower layers derived from the dual-phase solvent framework.

### 3.8. High-Speed Counter-Current Chromatography (HSCCC) Isolation Process

Prior to the addition of the stationary phase into the chromatographic column for distinct trials, the column underwent a thorough cleaning process utilizing methanol to rid it of any residual substances. In each separation venture, the multi-tiered column was initially charged to its full capacity with the upper phase, which served as the stationary phase. Subsequently, the lower phase, functioning as the mobile phase, was fed into the column at a flow rate of 1.5 mL/min while simultaneously rotating the device at a rate of 900 revolutions per minute.

As soon as the solvent front became visible and the system reached a hydrodynamic balance in a steady condition, the corresponding sample solutions were channeled into the separation column through the sample inlet, drawn from the sample loop. Data acquisition was initiated promptly following this injection procedure. Fraction collection was undertaken manually, guided by the readings on the chromatogram, followed by evaporation under diminished pressure conditions. The residues were then reconstituted in methanol, setting the stage for purity evaluations through UPLC analysis.

### 3.9. Determination of Target Constituents

The ^1^H NMR spectroscopic data were collected using a Bruker 600 MHz NMR spectrometry device with tetramethylsilane functioning as the intrinsic reference benchmark, all undertaken in a CDCl_3_ solvent setting.

### 3.10. DPPH Scavenging Activity Evaluation

Appropriate amounts of the three targets were prepared as sample solutions over a range of (0, 10, 20, 40, 80, 160 μg/mL). DPPH was prepared with ethanol to 25 μg/mL concentration. The 96-well plate was added with a ratio of 3:7 sample solution and DPPH solution and incubated for 30 min in the dark, followed by the determination of the absorbance at 517 nm on a microplate reader. As a positive standard control, ascorbic acid and rutin were employed. The rate of DPPH radical scavenging was estimated as follows:

DPPH scavenging rate (%) = [1 − (A/A_0_)] × 100%

where A and A_0_ were the experimental and blank groups’ absorbance, respectively.

## 4. Conclusions

In this study, the online HPLC-DPPH system was successfully utilized for the screening of antioxidants in *U. laetevirens* extracts. Through this system, three target compounds exhibiting significant antioxidant activity were identified. The utilization of ultrasonic-assisted extraction techniques along with HSCCC chromatography facilitated the isolation and purification of these target antioxidants.

Our research demonstrates that HSCCC combined with DPPH-HPLC is a powerful strategy for antioxidant active molecules’ screening and separation. This work not only provides robust evidence of the antioxidant properties of *U. laetevirens* extracts but also lays a foundational platform for the further development and utilization of these active compounds in the future. This endeavor opens a promising pathway in the exploration of natural antioxidants, presenting an opportunity to bolster the utilization of *U. laetevirens* extracts in pharmaceutical and nutraceutical applications.

## Figures and Tables

**Table 1:** Box–Behnken design matrix and experimental response.

			Factor 1	Factor 2	Factor 3	Response 1
Std	ID	Run	A: Ultrasonic Power	B: Ethanol Concentration	C: Liquid/Solid Ratio	Content of Targets
			W	%	1	%
17	13	1	300	60	20	58.2
15	13	2	300	60	20	62.8
12	12	3	300	70	30	58.05
5	5	4	200	60	10	51.9
1	1	5	200	50	20	45.32
8	8	6	400	60	30	58.19
4	4	7	400	70	20	60.51
13	13	8	300	60	20	61.89
11	11	9	300	50	30	52.42
10	10	10	300	70	10	56.23
7	7	11	200	60	30	48.86
16	13	12	300	60	20	59.55
6	6	13	400	60	10	55.59
9	9	14	300	50	10	48.56
2	2	15	400	50	20	46.39
3	3	16	200	70	20	50.01
14	13	17	300	60	20	61.49

**Table 2:** The analysis of variance.

Source	Sum of Squares	df	Mean Square	F-Value	*p*-Value
**Model**	498.47	9	55.39	17.06	0.0006
A-Ultrasonic power	75.58	1	75.58	23.28	0.0019
B-Ethanol concentration	128.88	1	128.88	39.7	0.0004
C-Liquid/solid ratio	3.43	1	3.43	1.06	0.3381
AB	22.23	1	22.23	6.85	0.0346
AC	7.95	1	7.95	2.45	0.1615
BC	1.04	1	1.04	0.3205	0.589
A^2^	114.04	1	114.04	35.13	0.0006
B^2^	106.29	1	106.29	32.74	0.0007
C^2^	15.96	1	15.96	4.92	0.0621
**Residual**	22.73	7	3.25		
Lack of Fit	8.74	3	2.91	0.8332	0.5413
Pure Error	13.99	4	3.5		
**Cor Total**	521.19	16			

**Table 3:** *K*-values of target compounds.

Solvent System	*K*_1_	*K*_2_	*K*_3_
4:1:5	0.13	7.82	9.19
3:1:5	0.16	6.15	8.32
2:1:5	0.20	5.85	7.02
3:2:5	0.32	3.11	5.21
2:3:5	0.58	1.73	2.56
1:4:5	0.79	0.88	1.43

## Data Availability

The data presented in this study are available on request from the corresponding author.

## Supplementary Materials

The following supporting information can be downloaded at: https://www.mdpi.com/article/10.3390/molecules28217332/s1, Figures S1–S6: the ^1^H and ^13^C NMR spectra of the three targets.

## Sample Availability

The samples are available from the authors.
