# Supplementary material for: Target-Guided Isolation and Purification of Antioxidants from Urtica laetevirens Maxim. by HSCCC Combined with Online DPPH-HPLC Analysis
Source: Molecules. 2023 Oct 29;28(21):7332. doi: 10.3390/molecules28217332 (PMC10650309; doi:10.3390/molecules28217332)
Supplement: Supplementary file 1 [file molecules-28-07332-s001.zip › molecules-2645422-supplementary.pdf]

Supporting Materials for

Target-guided isolation and purification of antioxidants from *Urtica laetevirens Maxim.* by HSCCC combined with online DPPH-HPLC analysis

Aijing Li[1,†], Mencuo La[1,†], Huichun Wang[1,*], Jianzhong Zhao[2], Yao Wang[1], Ruisha Mian[1], Fangfang He[1], Yuhan Wang[1], Tingqin Yang[1], Denglang Zou[1, 2, *]

[1] School of Life Science, Qinghai Normal University, Xining 810008, China

[2] Agricultural and Rural Science and Technology Guidance Development Service Center of Qinghai Province, Xining 810008, China

∗ Corresponding authors:

dlangzou@foxmail.com (D. L. Zou), whch_66@163.com (H. C. Wang)

[†] Aijing Li and Mencuo La contributed equally to this paper.

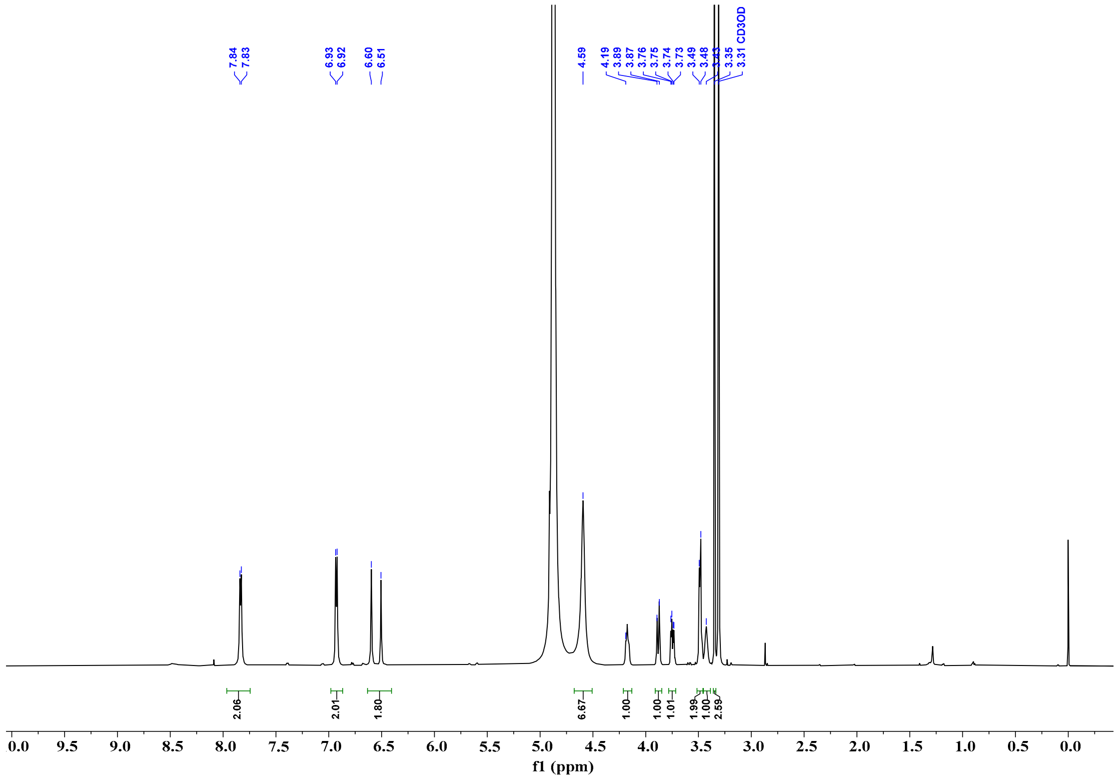

Figure S1. ^{1}H NMR spectrum of Isovitexin.

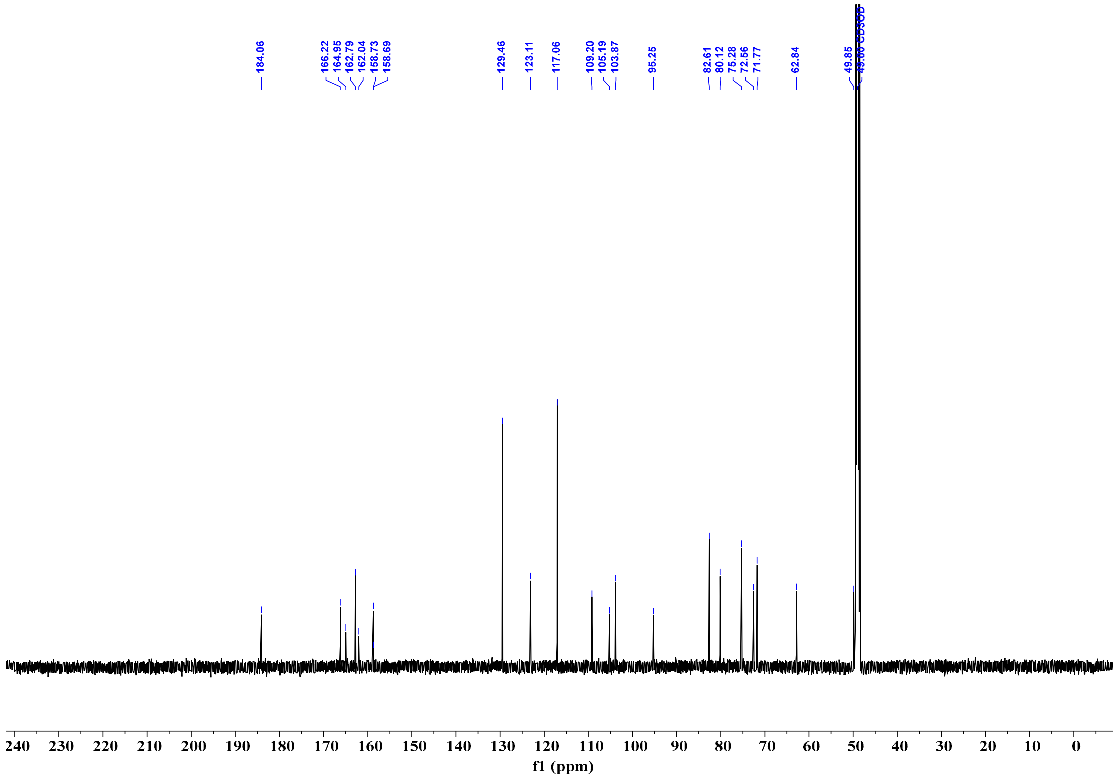

Figure S2. ^{13}C NMR spectrum of Isovitexin.

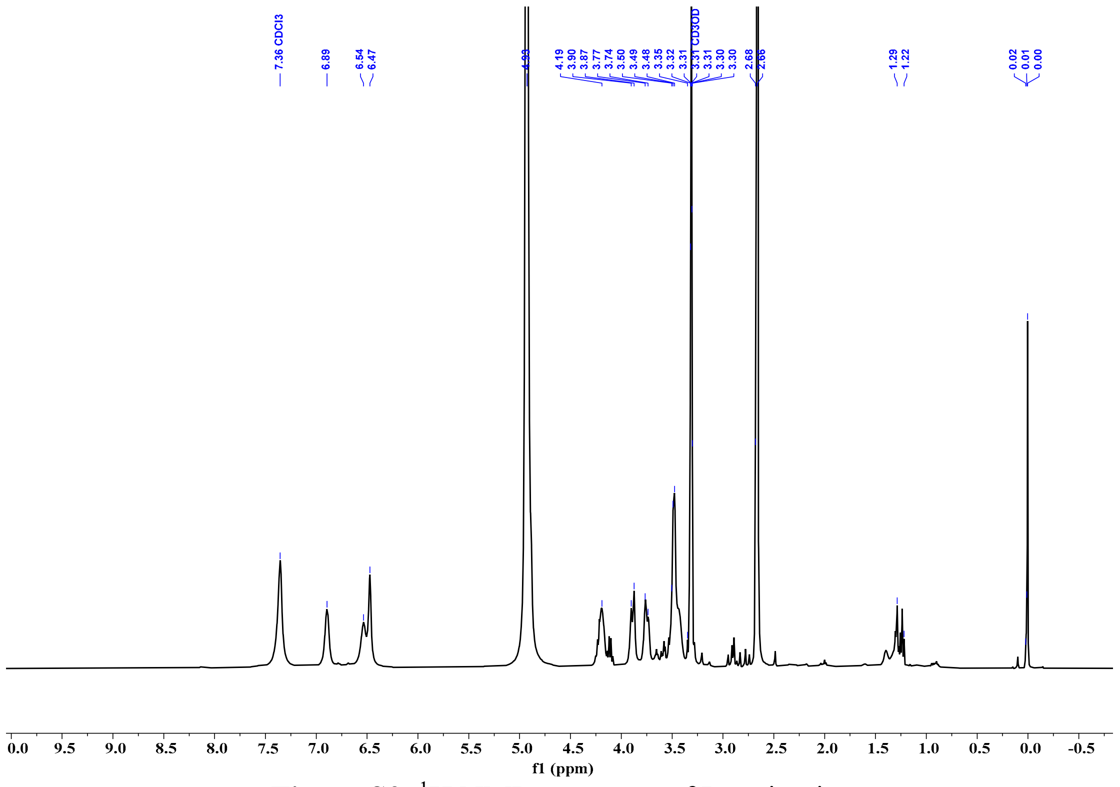

Figure S3. ^{1}H NMR spectrum of Isoorientin.

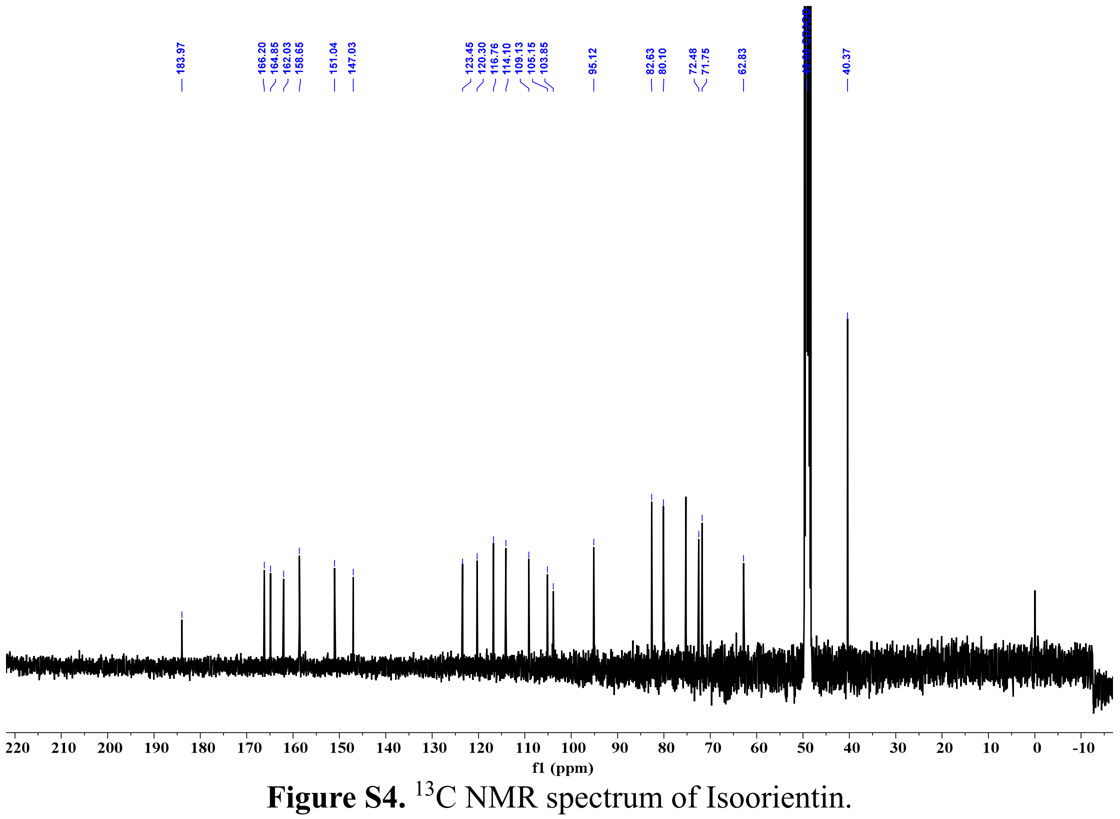

Figure S4. ^{13}C NMR spectrum of Isoorientin.

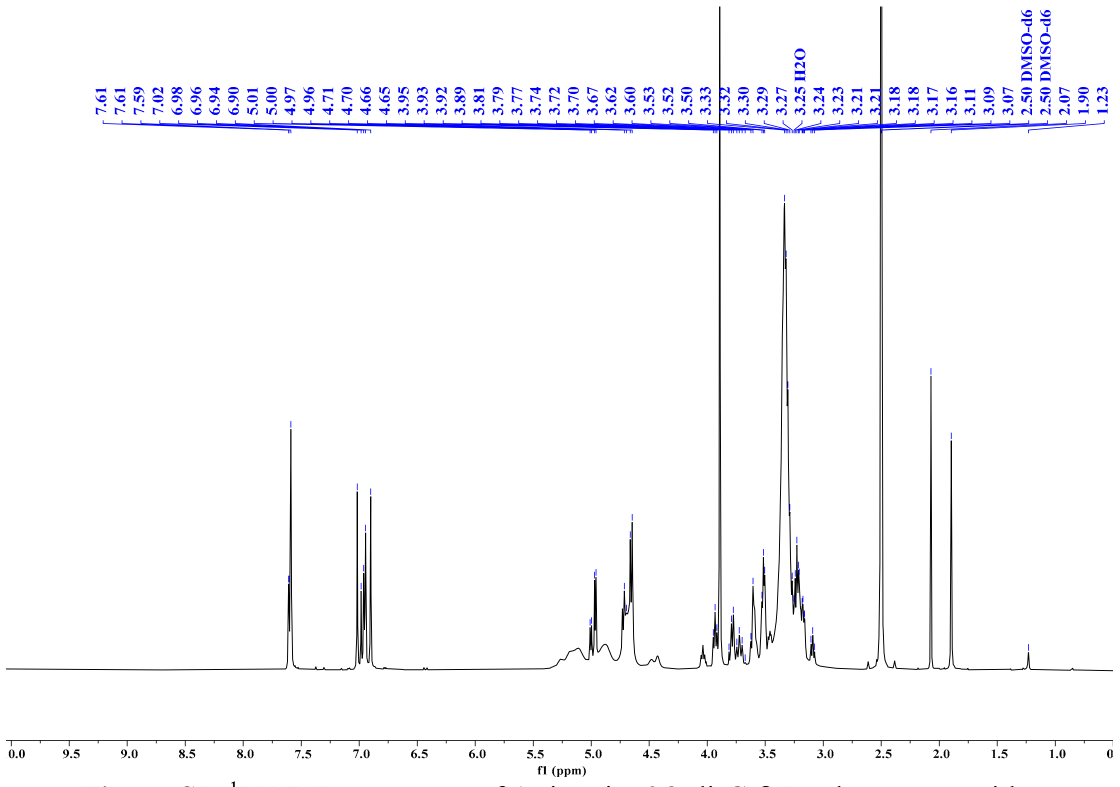

Figure S5. ^{1}H NMR spectrum of Apigenin-6,8-di-C-β-D-glucopyranoside.

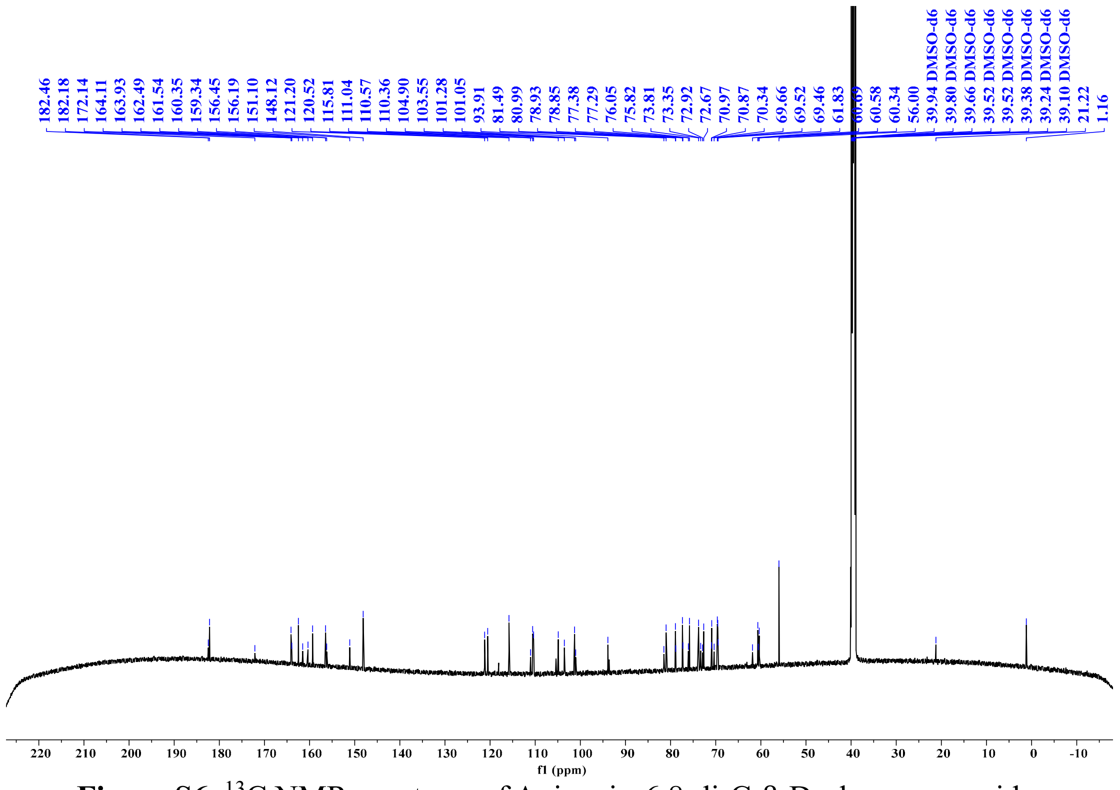

Figure S6. ^{13}C NMR spectrum of Apigenin-6,8-di-C-β-D-glucopyranoside.
